# Open Biology: overview for special issue on dynamics of protein fatty acylation

**DOI:** 10.1098/rsob.210228

**Published:** 2021-09-15

**Authors:** Marilyn D. Resh

**Affiliations:** Cell Biology Program, Memorial Sloan Kettering Cancer Center, 1275 York Avenue, Box 143, New York, NY 10075, USA

**Keywords:** fatty acylation, palmitoylation, zDHHC enzymes, MBOATs, signal transduction

## Abstract

Fatty acylation is a widespread form of protein modification that occurs on specific intracellular and secreted proteins. Beyond increasing hydrophobicity and the affinity of the modified protein for lipid bilayers, covalent attachment of a fatty acid exerts effects on protein localization, inter- and intramolecular interactions and signal transduction. As such, research into protein fatty acylation has been embraced by an extensive community of biologists. This special issue highlights advances at the forefront of the field, by focusing on two families of enzymes that catalyse post-translational protein fatty acylation, zDHHC palmitoyl acyltransferases and membrane-bound O-acyl transferases, and signalling pathways regulated by their fatty acylated protein substrates. The collected contributions catalogue the tremendous progress that has been made in enzyme and substrate identification. In addition, articles in this special issue provide insights into the pivotal functions of fatty acylated proteins in immune cell, insulin and EGF receptor-mediated signalling pathways. As selective inhibitors of protein fatty acyltransferases are generated, the future holds great promise for therapeutic targeting of fatty acyltransferases that play key roles in human disease.

## 1. Introduction

Covalent attachment of fatty acids to proteins is a form of lipid modification that occurs on thousands of proteins. Fatty acylation exerts pleiotropic effects on protein structure and function, influencing intracellular trafficking, protein–protein and protein–lipid interactions, protein stability and/or signalling functions [1,2]. The vast majority of protein fatty acylation occurs by thioester modification of cysteine residues with palmitate, a 16-carbon saturated fatty acid. These reactions, referred to as S-palmitoylation or S-acylation, are catalysed by the zDHHC family of palmitoyl acyltransferases (PATs). The human proteome encodes 23 zDHHC proteins, characterized by the presence of a conserved tetrapeptide Asp–His–His–Cys sequence within a cysteine-rich domain. It is well established that, for most zDHHC enzymes, catalysis proceeds via a two-step mechanism. First, palmitate is transferred from palmitoyl-CoA to the cysteine in the DHHC motif, then palmitate is transferred from the auto-palmitoylated enzyme to the recipient protein substrate [3,4]. However, we still do not fully understand how specific cysteine residues within a given protein are selected for acylation, and how specific proteins are selected for acylation by one or more PATs. These issues are addressed in several of the contributions in this special issue.

A second family of fatty acyltransferases, termed MBOAT (membrane-bound O-acyl transferase), catalyses the attachment of fatty acids to intracellular lipids and secreted proteins. Three MBOATs—Hhat (Hedgehog acyltransferase), Porcn (Porcupine) and GOAT (ghrelin O-acyltransferase)—comprise a subgroup that mediates fatty acylation of secreted signalling proteins. Hhat catalyses the attachment of palmitate to Hedgehog proteins, Porcn acylates Wnt proteins with palmitoleoate and GOAT links octanoate to the peptide hormone ghrelin. These reactions primarily occur on the luminal side of the ER membrane, although the recent finding that GOAT also resides at the plasma membrane allows for potential cell-surface re-acylation of ghrelin. In each case, fatty acylation is essential for the signalling function of the modified substrates. The challenges to understanding the molecular mechanisms of MBOAT-mediated fatty acylation reactions are at least 2-fold. First, Hhat, Porcn and GOAT are multipass transmembrane proteins whose complex structures and transmembrane domain topologies are just beginning to be elucidated. Second, there needs to be a mechanism to allow fatty acylation of proteins located in the ER lumen to occur, given that their fatty acyl-CoA substrates, which are not membrane permeable, are restricted to the cytosol.

While the enzymology of protein fatty acylation is fascinating on its own, an equally compelling issue is: what is the functional significance of attaching a fatty acid to a protein? An exciting aspect of this field is that fatty acylated proteins play pivotal roles in cellular signalling, examples of which are reviewed in this special issue. It is striking to view the myriad of cellular processes that are dependent on protein lipidation. Moreover, the reversible nature of some fatty acylation reactions adds another layer of complexity to these modifications. The thioester link between palmitate and S-acylated proteins can be broken by thioesterases [5], and O-acylation of Wnt proteins and ghrelin can be reversed by the extracellular deacylase Notum [6]. The balance between acylation and deacylation reactions thereby fine-tunes signalling pathway dynamics.

## 2. Scope and overview of the special issue

This special issue comprises nine contributions that examine a range of signalling pathways regulated by fatty acylated proteins as well as the fatty acyl transferases that modify these proteins. The contributions are grouped into three subject areas: MBOATs, zDHHCs and signalling pathways regulated by S-palmitoylated proteins.

### 2.1. Membrane-bound O-acyl transferases

Two reviews and a primary research paper address the structure and function of three enzymes that are MBOAT family members. The review by Resh summarizes our current knowledge of Hhat, including enzymatic assays, small molecule inhibitors and mechanisms involved in the recognition of protein and fatty acyl-CoA substrates [7]. Hhat catalyses N-palmitoylation, a reaction that occurs on the N-terminal Cys generated after removal of the signal sequence. *In vitro* assays that use N-terminal Hedgehog peptides as Hhat substrates have facilitated studies of Hhat enzymology as well as identification of small molecule Hhat inhibitors. Resh discusses the issue of transmembrane catalysis, citing evidence that Hhat contains an internal tunnel that allows access of cytosolic palmitoyl-CoA to the luminal side of the ER membrane for transfer to Hedgehog proteins. Similar intramembrane channels have been identified in structures of other MBOATs. New insights provided by three-dimensional structural studies of Hedgehog and its receptor, Patched1, illustrate how palmitoylation controls Hedgehog signalling. These studies have furthered our understanding of the role of activated Hedgehog signalling during development, and in lung, pancreatic and breast cancer in adults.

Porcn also uses a 16-carbon fatty acyl-CoA substrate, but in this case the fatty acid donor is the monounsaturated 16 : 1 palmitoleoyl-CoA. Porcn-mediated fatty acylation of Wnt proteins is required for Wnt secretion, binding to its co-receptor frizzled and signalling. To date, three-dimensional structural information has not been available for Porcn, although several transmembrane domain topology models of Porcn have been proposed. Burrus and co-workers [8] provide additional insight into the structure of Porcn using a combination of differential detergent solubilization and homology modelling. Cells expressing untagged or epitope-tagged Porcn constructs were treated with digitonin, which only solubilizes the plasma membrane, or Triton X-100, which solubilizes all cell membranes. Accessibility of either a homemade polyclonal antibody to Porcn or anti-Flag or anti-myc antibodies allowed the authors to assign the location of each epitope to the cytosol or the ER lumen. Additional refinement was provided by homology modelling to DltB, a bacterial MBOAT that shares only 15% identity to Porcn, along with molecular dynamics simulations. The authors propose a topology model for Porcn that contains nine transmembrane and two re-entrant membrane domains. The structure contains a funnel that could allow palmitoleoyl-CoA to shuttle from the cytoplasmic to the luminal side of the ER membrane. The conserved active site histidine sits at the apex of the funnel, and Wnt is proposed to access the active site from the ER lumen via insertion of a thumb-like projection surrounding the fatty acylated serine.

Hougland and co-workers [9] review the biochemistry and biology of ghrelin acylation by GOAT. Ghrelin is a 28 amino acid secreted peptide that is acylated with octanoate on Serine-3 by GOAT. This reaction is unique, as ghrelin is the only known octanoylated protein and the only known substrate for GOAT. Acylated ghrelin binds to the growth hormone secretagogue receptor, GHS-R1a, resulting in increased intracellular Ca++, growth hormone release and activation of a variety of signalling pathways, including AMPK, Akt/PI3 kinase, mTOR and MAPK. Elements required for substrate recognition by GOAT have been revealed by a predicted three-dimensional structure based on coevolutionary contact analysis [10]. GOAT contains an internal channel that passes through the enzyme from the cytoplasm to the ER, allowing entry of cytoplasmic octanoyl-CoA on one side and ghrelin from the other side of the membrane. An intriguing finding that GOAT also resides at the plasma membrane may enable ghrelin re-acylation and account for the ability of non-acylated (des-acyl)-ghrelin to signal. Peptidomimetics and bisubstrate mimetics that inhibit GOAT *in vitro* have been designed, with newer small molecule GOAT inhibitors showing promise for inhibiting ghrelin action *in vivo*.

### 2.2. zDHHC PATs: enzymology and structure

One of the major issues in the study of zDHHC enzymes is understanding how substrate recruitment and specificity are regulated. These key concepts are covered in the review by Malgapo & Linder [11]. Individual zDHHC enzymes can palmitoylate multiple protein substrates, an individual protein can often be palmitoylated by more than one zDHHC enzyme, and some proteins can be acylated with fatty acids other than palmitate. Three-dimensional structures of zDHHC enzymes have shed light on fatty acid chain length selectivity [12]. The size and shape of the hydrophobic cavity that binds the fatty acid influences which fatty acids are bound to and then transferred by an individual zDHHC to form an acylated protein substrate. Malgapo and Linder discuss the use of knockdown, knockout and overexpression of specific zDHHCs as well as mass spectrometry and quantitative proteomics to identify specific protein substrates acylated by a given zDHHC, and conversely to identify specific zDHHC(s) that acylate a particular S-acylated protein. The authors note the importance of considering potential effects of zDHHC depletion and overexpression on enzyme protein levels and intracellular localization, alterations of which could lead to misidentification of non-natural substrates. zDHHCs that contain additional sequences (SH3 and Akr domains, PDZ binding motifs) are highlighted, as these protein–protein interaction motifs play a role in enzyme–substrate recruitment. An additional twist comes from findings of enzyme crosstalk: some zDHHCs are themselves palmitoylated by another zDHHC, a process that has been shown to regulate substrate recruitment and enzyme activity [13].

Banerjee and co-workers [14] also address the issue of protein substrate engagement by zDHHC PATs. This laboratory was the first to report three-dimensional crystal structures of zDHHC enzymes [12]. Here, the authors review studies that have successfully reconstituted S-acylation *in vitro* with purified components. A key feature is an initial interaction of the protein substrate with the lipid bilayer. This is consistent with the location of the DHHC catalytic centre at the membrane–cytosol interface, and the findings that cysteine residues positioned near the membrane are often S-palmitoylated. The authors discuss the utility of using *in vitro* reconstitution assays to define the protein–protein interaction surface between an individual zDHHC enzyme and its substrates.

The redundancy built into the zDHHC family has made it difficult to identify specific small molecule inhibitors of individual enzymes. This problem has been addressed by Valdez Taubas and co-workers [15], who use a clever, elegant screening assay in yeast to identify zDHHC inhibitors. They employed a positive selection strategy using a yeast strain that grows only when palmitoylation is inhibited. A HIS3 reporter gene was placed under the control of a chimeric transcription factor (LexA-VP16) fused to Yck2, a substrate for palmitoylation by the PAT Akr1. The palmitoylated fusion protein localizes to the plasma membrane, preventing entry into the nucleus, and as a result, cells cannot grow in the absence of histidine. Screening of 3200 compounds using this system identified small molecules that inhibit Akr1. Moreover, the screening platform was adapted to include zDHHC20 and zDHHC21 enzymes and substrates. This innovative technology holds great promise for future identification of mammalian PAT inhibitors.

### 2.3. Regulation of signalling pathways by palmitoylated proteins

Three reviews in this special issue cover the wide-ranging roles that S-acylated proteins play in immune signalling, insulin signalling and EGF receptor-mediated signalling pathways. Hang and co-workers [16] provide an extensive compendium of S-palmitoylated proteins that function as immunity receptors, adapters and effectors. Proteins involved in adaptive immunity, such as the T-cell receptor co-receptors CD4 and CD8, Src family kinases, transmembrane adaptor proteins (LAT), as well as programmed cell death signalling molecules (FasL, Fas, PD-1, PD-L1) have been shown to be S-palmitoylated. Innate immunity signalling by Toll-like receptors, STING, NOD1/2, interferon and interferon effectors is also regulated by S-palmitoylation. Three overriding themes stand out. (i) S-palmitoylation generally serves as a positive regulator to stimulate signalling by the lipidated proteins. (ii) S-palmitoylation promotes association with lipid rafts, specialized subdomains of the plasma membrane enriched in cholesterol and phospholipids containing saturated fatty acid chains. (iii) Raft association enables the S-acylated proteins to interact with raft-associated receptors and signalling proteins, enhancing recruitment of downstream signalling molecules. The article also highlights several potential venues for therapeutic targeting of protein palmitoylation in autoimmune diseases such as Crohn's and inflammatory bowel disease.

Chamberlain *et al.* [17] focus on the roles that S-palmitoylation plays for proteins involved in insulin secretion and signalling. K+ and Ca++ channels are multi-subunit transmembrane proteins that regulate fusion of insulin-containing secretory granules with the plasma membrane. Several subunits of the K_ATP_, BK and voltage-gated Ca++ channels are S-palmitoylated and fatty acylation has been shown to regulate plasma membrane trafficking, subunit–subunit interaction and/or interaction of the acylated subunit with other membrane lipids (e.g. PIP2). As a result, changes in channel gating and electrical excitability lead to insulin secretion. After insulin release from pancreatic β-cells, binding to the insulin receptor triggers glucose uptake in fat and muscle cells. This occurs via translocation of insulin-responsive vesicles containing the GLUT4 glucose transporter to the plasma membrane, a process dependent on S-palmitoylated proteins such as SNAP23 and sortilin, as well as S-palmitoylation of GLUT4 itself. Attention has been focused on two PATs, zDHHC17 and zDHHC7, for their potential roles in regulating insulin secretion and GLUT4 acylation, respectively, and on zDHHC17 for its role in insulin signalling in adipocytes. However, the authors highlight the need for further information before specific PATs and protein substrates can be targeted therapeutically in insulin-dependent diseases such as diabetes.

Witze and co-workers [18] review the evidence that the EGF receptor (EGFR) is S-palmitoylated and discuss how EGFR signalling goes awry in tumour cells. Several cysteines in the C-terminal domain (CTD) of the EGFR are S-palmitoylated. Specifically, mutation of C1025 results in increased phosphorylation of EGFR and ERK1/2, suggesting a role for EGFR palmitoylation in an autoinhibitory function of the CTD. zDHHC20 is the likely PAT responsible for EGFR palmitoylation, and zDHHC20 depletion alters the duration and strength of downstream EGFR signalling in breast and lung cancer cell lines. Tissue-specific knockdown of zDHHC20 or introduction of a mutant EGFR (C1025A) in a genetically engineered mouse model of lung adenocarcinoma results in decreased tumour formation. The mechanism is probably through reduced PI3 K-Akt-c-Myc signalling, resulting in decreased cell proliferation. These findings may provide an impetus to develop zDHHC20 and other PAT inhibitors for potential therapeutic use in human tumours.

## 3. Concluding remarks and future directions

The contributions in this special issue illustrate the breadth of impact that fatty acylation exerts on protein function. As a biologist, you are guaranteed to find a fatty acylated protein involved in your specialized field of interest, including but not limited to immunology, neurobiology, developmental biology, metabolism and cancer biology. Technological advances have greatly enhanced our ability to identify fatty acylated proteins, and readers who are interested in determining if their favourite protein is S-palmitoylated are referred to the SwissPalm database [19]. However, detailed insight into the molecular mechanism of individual fatty acylation reactions remains challenging and is an ongoing endeavour. One of the most exciting advances in the field of fatty acylation has been the elucidation of three-dimensional structures of zDHHC and MBOAT enzymes, some of which were reported as this special issue was being assembled [12,20–23]. This information will not only shed light on the catalytic mechanisms employed by fatty acyltransferases but will also aid in the design and optimization of selective small molecule inhibitors to be used for therapeutic targeting in disease.

## Data accessibility

This article has no additional data.
